# Supplementary material for: The effect of resting tongue pressure, lip-cheek pressure, and interdental force on mandibular arch collapse and mesial molar drift: A preliminary 3D finite element study
Source: PLoS One. 2026 Oct 8;21(10):e0353500. doi: 10.1371/journal.pone.0353500 (PMC13649024; doi:10.1371/journal.pone.0353500)
Supplement: Supplementary file 1 [file pone.0353500.s002.docx]

Dear Dr. Alhendi and Dr. Abouelnour,

Thank you for your time and thoughtful comments. Your input has strengthened our paper, and we have done our best to address each point raised. As per your suggestions, the title has been changed, and limitations are now provided in a dedicated heading in the Results and Discussion Section: *Study Limitations and Future Directions*. This paper stands on its own merit, and we hope it will provide a step towards stimulating further research, and other researchers, to investigate the causes of orthodontic relapse and retention - an area of research that has remained relatively stagnant for many decades.

**Review Comments to the Author**

**Reviewer #1**:

NICE WRITTEN ARTICLE, IT FOCUS MORE ABOUT LOWER ARCH RETENTION AFTER ORTHODONTIC TREATMENT AND TRIGGER THE IMPOSTANCE OF FIXED RETENTION OR THE NEED FOR LONG RETENTION PERIOD AND FOLLOW UP. THANKS FOR YOUR EFFORTS

**OUR RESPONSE**: Thank you for your kind words. We have included modifications to the Results and Discussion Section:

“This study is important because a striking knowledge gap exists in our specialty: an incomplete understanding of post-alignment mandibular incisor crowding and mandibular anterior arch collapse. While significant advances have been made during the past century in orthodontic diagnosis and treatment, the equally important retention phase of care has remained relatively stagnant, with fixed or removable lifelong mechanical retention being the only guarantee for post-treatment dental alignment. We hope this study will provide a step towards stimulating further research with the long-term goal being stable post-treatment dental alignment tailored to each patient’s individual needs.”

**Reviewer #2**:

**I. Title Formulation & Conceptual Scope (Overclaiming)**

Observation: The use of the word "Etiology" in the manuscript title represents a significant overclaim. The collapse of the lower anterior arch is a complex, multifactorial phenomenon influenced by biological bone remodeling, occlusal forces, skeletal growth patterns, and soft tissue dynamics. The current study does not investigate comprehensive etiology; rather, it simulates the effects of three specific static mechanical loads on a simplified computer model.

Recommendation: To ensure scientific transparency and prevent misleading readers, the authors are strongly advised to revise the title to accurately reflect the true boundary of their investigation. A suggested title would be:

"The effect of resting tongue pressure, lip-cheek pressure, and interdental force on mandibular arch collapse and mesial molar drift: A preliminary 3D finite element study"

**OUR RESPONSE**: The title has been changed as per your suggestion:

"The effect of resting tongue pressure, lip-cheek pressure, and interdental force on mandibular arch collapse and mesial molar drift: A preliminary 3D finite element study"

**II. Materials & Methods Critique (Biomechanics & Modeling Assumptions):**

1.The authors explicitly state (lines 172–176) that the teeth were rigidly tied directly to the cancellous bone via Tie.

Critique: The PDL is the primary biological structure responsible for stress distribution, shock absorption, and the nonlinear viscoelastic movement of teeth under force. Omitting the PDL transforms the model from a biological tooth-movement simulation into a rigid structural deformation model. This significantly impairs the clinical external validity of the findings. This limitation must be explicitly highlighted and thoroughly discussed in a dedicated Study Limitations section.

**OUR RESPONSE**: Yes. Human PDL is an essential component of the human masticatory system. Ongoing studies continue to investigate the detailed mechanisms of PDL turnover during tooth movement including mechanocoupling, biomechanical coupling, cell-to-cell signaling, effector response, periodontal ligament viscoelasticity, hyalinization formation and resorption, alveolar bone deformation behavior, and mechanical stress regulation of stem/progenitor cell differentiation into osteoblast and osteoclast lineages, among others.

In truth, until an orthodontic tooth movement model incorporates all the above biology, it is fundamentally structural deformation in nature, falls short of fully capturing the exceedingly complex and incompletely understood dynamic resorption and deposition processes that occur clinically, and must operate within the confines of model assumptions and model complexity limitations. However, even as incomplete approximations, these models (including our present model) can offer valuable insights into the functioning of our masticatory system and stimulate further research.

What is known is that PDL hyalinization occurs when a load is applied to teeth which impedes capillary blood flow and that teeth will not move until hyalinized tissues are first resorbed. For our model we assumed that the thin hyalinized (PDL) tissue had been removed. Also, exhaustive attempts to include PDLs with an acceptable mesh density exceeded node limits for our academic license. We address the above under a new heading in the Results and Discussion Section: Study Limitations and Future Directions. The new entry is shown below:

“Study Limitations and Future Directions section:

● Roots of model teeth were connected directly to bone since our goal was to identify macroscopic teeth displacement patterns under (light) soft tissue loads, and our model was based upon the assumption that the thin, hyalinized PDL tissues had already been resorbed. Also, exhaustive attempts to include PDLs with an acceptable mesh density exceeded node limits for our academic license. Tie-constraining teeth roots directly to bone does not invalidate our conclusions."

2. Inaccuracy in Material Properties (Young's Modulus of Cancellous Bone):

Observation: In line 176, the elastic modulus (Young's\ Modulus) for cancellous bone is reported as 6\text{ kPa}.

Critique: This value appears to be a major typographical or input error. In established orthodontic FEA literature, the elastic modulus of mandibular cancellous bone consistently falls in the range of megapascals (100–1000\text{ MPa}), not kilopascals (kPa). The authors must carefully verify, correct, and justify this material input parameter, as an incorrect input invalidates the resulting stress/strain distribution values.

**OUR RESPONSE:** Yes, a smaller Young’s modulus of bone elasticity was intentionally chosen. As stated by Romanyk, FEA parameter values should be chosen to align with study goals.40 Our goal was to investigate teeth displacement patterns (relative displacements between teeth) when teeth were loaded with light soft tissue forces, and selecting a small Young’s modulus of bone elasticity from the literature resulted in ready identification of incisor displacement patterns.

In truth, if linearity of bone stiffness is assumed, then a stiffer / larger bone modulus would result in the same relative displacements between teeth (same displacement patterns) but with much smaller displacement magnitudes, making identification of these patterns much more difficult.

A statement highlighting this point has been included in the Results section:

"We wish to emphasize that a small Young’s modulus of bone elasticity was chosen since our goal was to investigate macroscopic teeth displacement patterns (relative displacements between teeth) when teeth were loaded with light soft tissue forces, and selecting a small Young’s modulus of bone elasticity from the literature resulted in ready identification of incisor displacement patterns. The absolute (millimetric) displacements should be interpreted as reflecting those patterns and not as in vivo displacements. If linearity of bone stiffness is assumed, then using a much larger bone modulus would result in the same relative displacements between teeth (same displacement patterns) but with much smaller displacement magnitudes, making identification of patterns difficult."

We also made an additional entry under our new heading in Results and Discussion: Study Limitations and Future Directions:

"● A small Young’s modulus of bone elasticity was chosen from the literature. The goal of our study was to investigate macroscopic teeth displacement patterns (relative displacements between teeth and not absolute millimetric displacements) when loaded with light soft tissue forces, and a small Young’s modulus of bone elasticity permitted ready visualization of these patterns. Under the assumption of linear bone elasticity, a stiffer bone modulus would result in the same relative displacements between teeth (same displacement patterns) albeit with much smaller displacement magnitudes, making identification of the patterns much more difficult. Using a small Young’s modulus of bone elasticity does not invalidate our conclusions."

3. Simplification of Transseptal Fibers (Interdental Force Simulation):

Observation: Interdental fiber forces were simulated by applying point-load traction vectors at the crown centroids.

Critique: In vivo, supracrestal transseptal fibers form a complex network operating above the alveolar crest. Applying a simplified point force at the crown centroid alters the resulting moment-to-force ratios (M/F) and tipping dynamics compared to real biological conditions.

**OUR RESPONSE**: This is an excellent point which we have taken the liberty of expanding upon under our new heading, Study Limitations and Future Directions.

"● Model teeth were loaded at centroids by lip / cheek pressure, tongue pressure, and transeptal fiber force. The literature contains many studies investigating lip / cheek pressure and tongue pressure magnitude but lacks studies detailing precisely where these pressures are applied to teeth crowns. Intuition suggests that these pressures are applied evenly over the buccal and lingual surfaces, but is that notion true? Or do lips apply pressure principally at incisal edges, centrally, or more gingivally? Are cheek pressures applied preferentially to maxillary posterior teeth and tongue pressures applied preferentially to mandibular posterior teeth, or are all pressures applied equally to both arches? Is greater pressure applied at the canines (cornerstones of the arch) compared to other teeth? Certainly, soft tissue pressure distribution must diminish as teeth crowns curve inward at their interproximal contacts but by how much and where? Answers to these questions fall within the scope of future clinical studies.

The difficulties in modeling interdental (transeptal fiber) forces are even more challenging. The literature contains only one study, a study we conducted,24 which estimates interdental contact force magnitude, and we only estimated this force between first permanent molars and second premolars. It is doubtful that the force generated by transeptal fibers between other teeth is the same, and further study is warranted. However, for this model we used the only data that was available.

In terms of interdental force location, since transeptal fibers contact adjoining teeth above the alveolar crest (connecting cementum of one tooth to cementum of adjacent teeth), applying this force at a centroid will affect the estimated moment-to-force (M/F) ratio and tipping dynamics relative to in vivo. A complication lies in the fact that mesial and distal cementoenamel junction contours arc occlusally (towards the centroid) on most mandibular teeth, making precise load location exceedingly complex.

For the above reasons, using the pressure / force data available and applying these loads centrally was a reasonable assumption but points to the fact that significant future clinical study is warranted to refine modeling."

4.The current model represents an isolated lower arch in space. Simulating occlusal forces, deep bite, or intercuspation cannot be performed on this isolated single-arch architecture; it necessitates a dual-arch model (Maxilla\ and\ Mandible) with complex 3D surface-to-surface contact formulations. The authors should clarify the structural inability of the current model to directly accommodate these complex variables without a complete redesign.

**OUR RESPONSE**: This is an excellent point which underscores the complexity of our masticatory system and the interrelationship between the jaws. We agree wholeheartedly that the maxillary arch probably (intuitively) plays a role in mandibular anterior crowding and arch collapse.

We have begun a follow-up thesis project which has added the six maxillary anterior teeth to the model (and includes complex 3D surface-to-surface contact formulations) to study the effects of overbite increase on the mandibular anterior arch. However, we agree unreservedly that a complete model redesign will indeed be necessary to study the effects of occlusal function.

When we began the present study, we were led by the words of Dr. William Proffit who stated that the major primary factors affecting dental equilibrium (or position imbalance and relapse) appear to be resting pressures of tongue and lips plus forces created within the periodontal tissues. The present, preliminary study is only one small, first step which corroborates Dr. Proffit’s thoughts and encourages future FEA relapse research. Beginning with the isolated mandibular arch was a reasonable first step. To clarify this, we made an additional entry under our new heading in Results and Discussion: Study Limitations and Future Directions:

"● The present model represents an isolated mandible in space. Intuition suggests that occlusal loading from the maxillary arch probably plays some role in mandibular anterior arch changes, and a complete model redesign will be needed to study occlusal loading effects. By adding six maxillary anterior teeth to the present model (and including complex 3D surface-to-surface contact formulations) we have initiated a limited thesis study of the effects of anterior overbite increase on the mandibular anterior arch.

Dr. William Proffit stated that the major primary factors affecting dental equilibrium (or position imbalance and relapse) appears to be resting pressures of tongue and lips plus forces created within the periodontal tissues. The present, preliminary study is only one small, first step which corroborates Dr. Proffit’s thoughts and encourages future FEA relapse research. Beginning with the isolated mandible was a reasonable first step."

Thank you Dr. Esam Abdullah Alhendi for your comments and suggestions. They have strengthened our manuscript.
