# Supplementary material for: The effect of resting tongue pressure, lip-cheek pressure, and interdental force on mandibular arch collapse and mesial molar drift: A preliminary 3D finite element study
Source: PLoS One. 2026 Oct 8;21(10):e0353500. doi: 10.1371/journal.pone.0353500 (PMC13649024; doi:10.1371/journal.pone.0353500)
Supplement: Peer review history

# Peer review: Decision Letter 0

**DOI:** 10.1371/journal.pone.0353500.r001

**Authors:** Tanay Chaubal

6 Aug 2026

Dear Dr.  Marshall

Thank you for submitting your manuscript to PLOS ONE. After careful consideration, we feel that it has merit but does not fully meet PLOS ONE’s publication criteria as it currently stands. Therefore, we invite you to submit a revised version of the manuscript that addresses the points raised during the review process.

==============================

**ACADEMIC EDITOR: I thank you for the time and effort put in preparing and submitting the manuscript. However, there are a few queries raised by the reviewers' which need to be addressed to make the manuscript more robust.**

==============================

Please submit your revised manuscript by Sep 20 2026 11:59PM. If you will need more time than this to complete your revisions, please reply to this message or contact the journal office at plosone@plos.org. When you're ready to submit your revision, log on to https://www.editorialmanager.com/pone/ and select the 'Submissions Needing Revision' folder to locate your manuscript file.

- A letter that responds to each point raised by the academic editor and reviewer(s). You should upload this letter as a separate file labeled 'Response to Reviewers'.
- A marked-up copy of your manuscript that highlights changes made to the original version. You should upload this as a separate file labeled 'Revised Manuscript with Track Changes'.
- An unmarked version of your revised paper without tracked changes. You should upload this as a separate file labeled 'Manuscript'.

If you would like to make changes to your financial disclosure, please include your updated statement in your cover letter. Guidelines for resubmitting your figure files are available below the reviewer comments at the end of this letter.

If applicable, we recommend that you deposit your laboratory protocols in protocols.io to enhance the reproducibility of your results. Protocols.io assigns your protocol its own identifier (DOI) so that it can be cited independently in the future. For instructions see: https://journals.plos.org/plosone/s/submission-guidelines#loc-laboratory-protocols. Additionally, PLOS ONE offers an option for publishing peer-reviewed Lab Protocol articles, which describe protocols hosted on protocols.io. Read more information on sharing protocols at https://plos.org/protocols?utm_medium=editorial-email&utm_source=authorletters&utm_campaign=protocols.

As the corresponding author, your ORCID iD is verified in the submission system and will appear in the published article. PLOS supports the use of ORCID, and we encourage all coauthors to register for an ORCID iD and use it as well. Please encourage your coauthors to verify their ORCID iD within the submission system before final acceptance, as unverified ORCID iDs will not appear in the published article. *Only*  the individual author can complete the verification step; PLOS staff *cannot*  verify ORCID iDs on behalf of authors.

We look forward to receiving your revised manuscript.

Kind regards,

Tanay Chaubal

Academic Editor

PLOS One

Journal Requirements:

When submitting your revision, we need you to address these additional requirements.

1.Please ensure that your manuscript meets PLOS ONE's style requirements, including those for file naming. The PLOS ONE style templates can be found at

https://journals.plos.org/plosone/s/file?id=wjVg/PLOSOne_formatting_sample_main_body.pdf and

https://journals.plos.org/plosone/s/file?id=ba62/PLOSOne_formatting_sample_title_authors_affiliations.pdf

2. Please provide a complete Data Availability Statement in the submission form, ensuring you include all necessary access information or a reason for why you are unable to make your data freely accessible. If your research concerns only data provided within your submission, please write "All data are in the manuscript and/or supporting information files" as your Data Availability Statement.

3. We notice that your supplementary figures are uploaded with the file type 'Figure'. Please amend the file type to 'Supporting Information'. Please ensure that each Supporting Information file has a legend listed in the manuscript after the references list.

4. If the reviewer comments include a recommendation to cite specific previously published works, please review and evaluate these publications to determine whether they are relevant and should be cited. There is no requirement to cite these works unless the editor has indicated otherwise.

[Note: HTML markup is below. Please do not edit.]

Reviewer's Responses to Questions

**Comments to the Author**

1. Is the manuscript technically sound, and do the data support the conclusions?

Reviewer #1: Yes

Reviewer #2: Yes

**********

2. Has the statistical analysis been performed appropriately and rigorously?

Reviewer #1: Yes

Reviewer #2: I Don't Know

**********

3. Have the authors made all data underlying the findings in their manuscript fully available?

The PLOS Data policy

Reviewer #1: Yes

Reviewer #2: Yes

**********

4. Is the manuscript presented in an intelligible fashion and written in standard English?

Reviewer #1: Yes

Reviewer #2: Yes

**********

Reviewer #1: NICE WRITTEN ARTICLE, IT FOCUS MORE ABOUT LOWER ARCH RETENTION AFTER ORTHODONTIC TREATMENT AND TRIGGER THE IMPOSTANCE OF FIXED RETENTION OR THE NEED FOR LONG RETENTION PERIOD AND FOLLOW UP. THANKS FOR YOUR EFFORTS.

Reviewer #2: I would like to express my sincere gratitude for the kind invitation to review this manuscript. After carefully reading and evaluating the manuscript, I identified several important scientific and methodological issues that warrant careful consideration and appropriate revision. Addressing these points would, in my opinion, contribute significantly to improving the quality, clarity, and scientific rigor of the manuscript.

I. Title Formulation & Conceptual Scope (Overclaiming)

Observation: The use of the word "Etiology" in the manuscript title represents a significant overclaim. The collapse of the lower anterior arch is a complex, multifactorial phenomenon influenced by biological bone remodeling, occlusal forces, skeletal growth patterns, and soft tissue dynamics. The current study does not investigate comprehensive etiology; rather, it simulates the effects of three specific static mechanical loads on a simplified computer model.

Recommendation: To ensure scientific transparency and prevent misleading readers, the authors are strongly advised to revise the title to accurately reflect the true boundary of their investigation. A suggested title would be:

"The effect of resting tongue pressure, lip-cheek pressure, and interdental force on mandibular arch collapse and mesial molar drift: A preliminary 3D finite element study"

II. Materials & Methods Critique (Biomechanics & Modeling Assumptions:

1.The authors explicitly state (lines 172–176) that the teeth were rigidly tied directly to the cancellous bone via Tie

Critique: The PDL is the primary biological structure responsible for stress distribution, shock absorption, and the nonlinear viscoelastic movement of teeth under force. Omitting the PDL transforms the model from a biological tooth-movement simulation into a rigid structural deformation model. This significantly impairs the clinical external validity of the findings. This limitation must be explicitly highlighted and thoroughly discussed in a dedicated Study Limitations section

2. Inaccuracy in Material Properties (Young's Modulus of Cancellous Bone):

Observation: In line 176, the elastic modulus (Young's\ Modulus) for cancellous bone is reported as 6\text{ kPa}.

Critique: This value appears to be a major typographical or input error. In established orthodontic FEA literature, the elastic modulus of mandibular cancellous bone consistently falls in the range of megapascals (100–1000\text{ MPa}), not kilopascals (kPa). The authors must carefully verify, correct, and justify this material input parameter, as an incorrect input invalidates the resulting stress/strain distribution values.

3. Simplification of Transseptal Fibers (Interdental Force Simulation):

Observation: Interdental fiber forces were simulated by applying point-load traction vectors at the crown centroids.

Critique: In vivo, supracrestal transseptal fibers form a complex network operating above the alveolar crest. Applying a simplified point force at the crown centroid alters the resulting moment-to-force ratios (M/F) and tipping dynamics compared to real biological conditions

4.The current model represents an isolated lower arch in space. Simulating occlusal forces, deep bite, or intercuspation cannot be performed on this isolated single-arch architecture; it necessitates a dual-arch model (Maxilla\ and\ Mandible) with complex 3D surface-to-surface contact formulations. The authors should clarify the structural inability of the current model to directly accommodate these complex variables without a complete redesign

**********

what does this mean?). If published, this will include your full peer review and any attached files.

If you choose “no”, your identity will remain anonymous but your review may still be made public.

**Do you want your identity to be public for this peer review?**  For information about this choice, including consent withdrawal, please see our Privacy Policy

Reviewer #1: **Yes:**  Prof. Dr. Ahmed Abouelnour

Reviewer #2: **Yes:**  Esam Abdullah Alhendi

**********

[NOTE: If reviewer comments were submitted as an attachment file, they will be attached to this email and accessible via the submission site. Please log into your account, locate the manuscript record, and check for the action link "View Attachments". If this link does not appear, there are no attachment files.]

To ensure your figures meet our technical requirements, please review our figure guidelines: https://journals.plos.org/plosone/s/figures

You may also use PLOS’s free figure tool, NAAS, to help you prepare publication quality figures: https://journals.plos.org/plosone/s/figures#loc-tools-for-figure-preparation.

NAAS will assess whether your figures meet our technical requirements by comparing each figure against our figure specifications.

# Author comment: Author response to Decision Letter 1

**DOI:** 10.1371/journal.pone.0353500.r002

4 Sep 2026

Dear Academic Editor Tanay Chaubal,

Thank you for your kind consideration of our original manuscript and your instructions for its improvement. We have endeavored to address the requirements necessary for our revised manuscript. I provide a summary of that effort below. Our responses to your requirements are found below each requirement.

ACADEMIC EDITOR COMMENTS AND REQUIREMENTS:

Please include the following items (1-6) when submitting your revised manuscript:

1. A letter that responds to each point raised by the academic editor and reviewer(s). You should upload this letter as a separate file labeled 'Response to Reviewers'.

OUR RESPONSE: We have uploaded a Response to Reviewers’ letter that addresses each point made by the reviewers. It was uploaded under Select Item Type -> Response to Reviewers, labeled Response to Reviewers. We have duplicated this response below under the heading REVIEWER COMMENTS TO AUTHOR.

2. A marked-up copy of your manuscript that highlights changes made to the original version. You should upload this as a separate file labeled 'Revised Manuscript with Track Changes'.

OUR RESPONSE: We have uploaded a separate file marked with our changes to our original submission using Microsoft Word “Track Changes” and under Select Item Type -> Revised Manuscript with Track Changes, labeled “Revised Manuscript with Track Changes”.

3. An unmarked version of your revised paper without tracked changes. You should upload this as a separate file labeled 'Manuscript'.

OUR RESPONSE: We have uploaded a separate file with our changes to our original submission that is unmarked under Select Item Type -> Manuscript, labeled “Revised manuscript clean copy”.

4. If you would like to make changes to your financial disclosure, please include your updated statement in your cover letter. Guidelines for resubmitting your figure files are available below the reviewer comments at the end of this letter.

OUR RESPONSE: We have no changes to our financial disclosure. We have followed the guidelines for resubmitting our figures using the separate software NAAS. For the revision, we removed our original figures and uploaded NAAS approved figures.

5. If applicable, we recommend that you deposit your laboratory protocols in protocols.io to enhance the reproducibility of your results. Protocols.io assigns your protocol its own identifier (DOI) so that it can be cited independently in the future. For instructions see: https://journals.plos.org/plosone/s/submission-guidelines#loc-laboratory-protocols. Additionally, PLOS ONE offers an option for publishing peer-reviewed Lab Protocol articles, which describe protocols hosted on protocols.io. Read more information on sharing protocols at https://plos.org/protocols?utm_medium=editorial-email&utm_source=authorletters&utm_campaign=protocols.

OUR RESPONSE: Our study does not have a laboratory protocol.

6. As the corresponding author, your ORCID iD is verified in the submission system and will appear in the published article. PLOS supports the use of ORCID, and we encourage all coauthors to register for an ORCID iD and use it as well. Please encourage your coauthors to verify their ORCID iD within the submission system before final acceptance, as unverified ORCID iDs will not appear in the published article. Only the individual author can complete the verification step; PLOS staff cannot verify ORCID iDs on behalf of authors.

OUR RESPONSE: Thank you for this information.

Journal Requirements: When submitting your revision, we need you to address these additional requirements .

1.Please ensure that your manuscript meets PLOS ONE's style requirements, including those for file naming. The PLOS ONE style templates can be found at https://journals.plos.org/. . . . . . /PLOSOne_formatting_sample_main_body.pdf and https://journals.plos.org/. . . . . ./PLOSOne_formatting_sample_title_authors_affiliations.pdf.

OUR RESPONSE: We carefully reviewed these PLOS ONE formatting samples to ensure our revised manuscript meets the journal’s style requirements, including file naming conventions.

2. Please provide a complete Data Availability Statement in the submission form, ensuring you include all necessary access information or a reason for why you are unable to make your data freely accessible. If your research concerns only data provided within your submission, please write "All data are in the manuscript and/or supporting information files" as your Data Availability Statement.

OUR RESPONSE: We apologize for our previous confusion on this point. We have uploaded under Select Item Type -> Supporting Information, an Excel spreadsheet that provides all data obtained in our experiments and used in compiling our manuscript. As per your instructions, our Data Availability Statement reads: "All data are in the manuscript and/or supporting information files.”

3. We notice that your supplementary figures are uploaded with the file type 'Figure'. Please amend the file type to 'Supporting Information'. Please ensure that each Supporting Information file has a legend listed in the manuscript after the references list.

OUR RESPONSE: We apologize for our confusion on this point. All of our 22 figures are noted in figure captions in the body of the manuscript and referred to as figures in the text. We do not understand how the use of supplementary figures applies to our manuscript. We are happy to abide by this requirement. Our mistake may be that we are referencing our previous PLOS ONE publication as a guide to how figures in the manuscript body and figures in supporting information are handled. Thank you in advance for helping our understanding of this point.

4. If the reviewer comments include a recommendation to cite specific previously published works, please review and evaluate these publications to determine whether they are relevant and should be cited. There is no requirement to cite these works unless the editor has indicated otherwise.

OUR RESPONSE: We did not receive specific reviewer comments that additional citations were needed.

REVIEWER COMMENTS TO AUTHOR

Reviewer #1:

NICE WRITTEN ARTICLE, IT FOCUS MORE ABOUT LOWER ARCH RETENTION AFTER ORTHODONTIC TREATMENT AND TRIGGER THE IMPOSTANCE OF FIXED RETENTION OR THE NEED FOR LONG RETENTION PERIOD AND FOLLOW UP. THANKS FOR YOUR EFFORTS.

OUR RESPONSE: We thank you, Dr. Abouelnour, for your kind words. We have included modifications to the Discussion paragraph:

"This study is important because a striking knowledge gap exists in our specialty: an incomplete understanding of post-alignment mandibular incisor crowding and mandibular anterior arch collapse. While significant advances have been made during the past century in orthodontic diagnosis and treatment, the equally important retention phase of care has remained relatively stagnant, with fixed or removable lifelong mechanical retention being the only guarantee for post-treatment dental alignment. We hope this study will provide a step towards stimulating further research with the long-term goal being stable post-treatment dental alignment tailored to each patient’s individual needs."

Reviewer #2:

I. Title Formulation & Conceptual Scope (Overclaiming)

Observation: The use of the word "Etiology" in the manuscript title represents a significant overclaim. The collapse of the lower anterior arch is a complex, multifactorial phenomenon influenced by biological bone remodeling, occlusal forces, skeletal growth patterns, and soft tissue dynamics. The current study does not investigate comprehensive etiology; rather, it simulates the effects of three specific static mechanical loads on a simplified computer model.

Recommendation: To ensure scientific transparency and prevent misleading readers, the authors are strongly advised to revise the title to accurately reflect the true boundary of their investigation. A suggested title would be:

"The effect of resting tongue pressure, lip-cheek pressure, and interdental force on mandibular arch collapse and mesial molar drift: A preliminary 3D finite element study"

OUR RESPONSE: The title has been changed as per your suggestion:

"The effect of resting tongue pressure, lip-cheek pressure, and interdental force on mandibular arch collapse and mesial molar drift: A preliminary 3D finite element study"

II. Materials & Methods Critique (Biomechanics & Modeling Assumptions):

1.The authors explicitly state (lines 172–176) that the teeth were rigidly tied directly to the cancellous bone via Tie

Critique: The PDL is the primary biological structure responsible for stress distribution, shock absorption, and the nonlinear viscoelastic movement of teeth under force. Omitting the PDL transforms the model from a biological tooth-movement simulation into a rigid structural deformation model. This significantly impairs the clinical external validity of the findings. This limitation must be explicitly highlighted and thoroughly discussed in a dedicated Study Limitations section.

OUR RESPONSE: Yes. Human PDL is an essential component of the human masticatory system. Ongoing studies continue to investigate the detailed mechanisms of PDL turnover during tooth movement including mechanocoupling, biomechanical coupling, cell-to-cell signaling, effector response, periodontal ligament viscoelasticity, hyalinization formation and resorption, alveolar bone deformation behavior, and mechanical stress regulation of stem/progenitor cell differentiation into osteoblast and osteoclast lineages, among others.

In truth, until an orthodontic tooth movement model incorporates all the above biology, it is fundamentally structural deformation in nature, falls short of fully capturing the exceedingly complex and incompletely understood dynamic resorption and deposition processes that occur clinically, and must operate within the confines of model assumptions and model complexity limitations. However, even as incomplete approximations, these models (including our present model) can offer valuable insights into the functioning of our masticatory system and stimulate further research.

What is known is that PDL hyalinization occurs when a load is applied to teeth which impedes capillary blood flow and that teeth will not move until hyalinized tissues are first resorbed. For our model we assumed that the thin hyalinized (PDL) tissue had been removed. Also, exhaustive attempts to include PDLs with an acceptable mesh density exceeded node limits for our academic license. We address the above under a new heading in the Results and Discussion Section: Study Limitations and Future Directions. The new entry is shown below:

Study Limitations and Future Directions section:

"● Roots of model teeth were connected directly to bone since our goal was to identify macroscopic teeth displacement patterns under (light) soft tissue loads, and our model was based upon the assumption that the thin, hyalinized PDL tissues had already been resorbed. Also, exhaustive attempts to include PDLs with an acceptable mesh density exceeded node limits for our academic license. Tie-constraining teeth roots directly to bone does not invalidate our conclusions."

2. Inaccuracy in Material Properties (Young's Modulus of Cancellous Bone):

Observation: In line 176, the elastic modulus (Young's\ Modulus) for cancellous bone is reported as 6\text{ kPa}.

Critique: This value appears to be a major typographical or input error. In established orthodontic FEA literature, the elastic modulus of mandibular cancellous bone consistently falls in the range of megapascals (100–1000\text{ MPa}), not kilopascals (kPa). The authors must carefully verify, correct, and justify this material input parameter, as an incorrect input invalidates the resulting stress/strain distribution values.

OUR RESPONSE: Yes, a smaller Young’s modulus of bone elasticity was intentionally chosen. As stated by Romanyk, FEA parameter values should be chosen to align with study goals.40 Our goal was to investigate teeth displacement patterns (relative displacements between teeth) when teeth were loaded with light soft tissue forces, and selecting a small Young’s modulus of bone elasticity from the literature resulted in ready identification of incisor displacement patterns.

In truth, if linearity of bone stiffness is assumed, then a stiffer / larger bone modulus would result in the same relative displacements between teeth (same displacement patterns) but with much smaller displacement magnitudes, making identification of these patterns much more difficult.

A statement highlighting this point has been included in the Results section:

"We wish to emphasize that a small Young’s modulus of bone elasticity was chosen since our goal was to investigate macroscopic teeth displacement patterns (relative displacements between teeth) when teeth were loaded with light soft tissue forces, and selecting a small Young’s modulus of bone elasticity from the literature resulted in ready identification of incisor displacement patterns. The absolute (millimetric) displacements should be interpreted as reflecting those patterns and not as in vivo displacements. If linearity of bone stiffness is assumed, then using a much larger bone modulus would result in the same relative displacements between teeth (same displacement patterns) but with much smaller displacement magnitudes, making identification of patterns difficult."

We also made an additional entry under our new heading in Results and Discussion: Study Limitations and Future Directions:

"● A small Young’s modulus of bone elasticity was chosen from the literature. The goal of our study was to investigate macroscopic teeth displacement patterns (relative displacements between teeth and not absolute millimetric displacements) when loaded with light soft tissue forces, and a small Young’s modulus of bone elasticity permitted ready visualization of these patterns. Under the assumption of linear bone elasticity, a stiffer bone modulus would result in the same relative displacements between teeth (same displacement patterns) albeit with much smaller displacement magnitudes, making identification of the patterns much more difficult. Using a small Young’s modulus of bone elasticity does not invalidate our conclusions."

3. Simplification of Transseptal Fibers (Interdental Force Simulation):

Observation: Interdental fiber forces were simulated by applying point-load traction vectors at the crown centroids.

Critique: In vivo, supracrestal transseptal fibers form a complex network operating above the alveolar crest. Applying a simplified point force at the crown centroid alters the resulting moment-to-force ratios (M/F) and tipping dynamics compared to real biological conditions.

OUR RESPONSE: This is an excellent point which we have taken the liberty of expanding upon under our new heading, Study Limitations and Future Directions.

"● Model teeth were loaded at centroids by lip / cheek pressure, tongue pressure, and transeptal fiber force.

The literature contains many studies investigating lip / cheek pressure and tongue pressure magnitude but lacks studies detailing precisely where these pressures are applied to teeth crowns. Intuition suggests that these pressures are applied evenly over the buccal and lingual surfaces, but is that notion true? Or do lips apply pressure principally at incisal edges, centrally, or more gingivally? Are cheek pressures applied preferentially to maxillary posterior teeth and tongue pressures applied preferentially to mandibular posterior teeth, or are all pressures applied equally to both arches? Is greater pressure applied at the canines (cornerstones of the arch) compared to other teeth? Certainly, soft tissue pressure distribution must diminish as teeth crowns curve inward at their interproximal contacts but by how much and where? Answers to these questions fall within the scope of future clinical studies.

The difficulties in modeling interdental (transeptal fiber) forces are even more challenging. The literature contains only one study, a study we conducted,24 which estimates interdental contact force magnitude, and we only estimated this force between first permanent molars and second premolars. It is doubtful that the force generated by transeptal fibers between other teeth is the same, and further study is warranted. However, for this model we used the only data that was available.

In terms of interdental force location, since transeptal fibers contact adjoining teeth above the alveolar crest (connecting cementum of one tooth to cementum of adjacent teeth), applying this force at a centroid will affect the estimated moment-to-force (M/F) ratio and tipping dynamics relative to in vivo. A complication lies in the fact that mesial and distal cementoenamel junction contours arc occlusally (towards the centroid) on most mandibular teeth, making precise load location exceedingly complex.

For the above reasons, using the pressure / force data available and applying these loads centrally was a reasonable assumption but points to the fact that significant future clinical study is warranted to refine modeling."

4.The current model represents an isolated lower arch in space. Simulating occlusal forces, deep bite, or intercuspation cannot be performed on this isolated single-arch architecture; it necessitates a dual-arch model (Maxilla\ and\ Mandible) with complex 3D surface-to-surface contact formulations. The authors should clarify the structural inability of the current model to directly accommodate these complex variables without a complete redesign.

OUR RESPONSE: This is an excellent point which underscores the complexity of our masticatory system and the interrelationship between the jaws. We agree wholeheartedly that the maxillary arch probably (intuitively) plays a role in mandibular anterior crowding and arch collapse.

We have begun a follow-up thesis project which has added the six maxillary anterior teeth to the model (and includes complex 3D surface-to-surface contact formulations) to study the effects of overbite increase on the mandibular anterior arch. However, we agree unreservedly that a complete model redesign will indeed be necessary to study the effects of occlusal function.

When we began the present study, we were led by the words of Dr. William Proffit who stated that the major primary factors affecting dental equilibrium (or position imbalance and relapse) appear to be resting pressures of tongue and lips plus forces created within the periodontal tissues. The present, preliminary study is only one small, first step which corroborates Dr. Proffit’s thoughts and encourages future FEA relapse research. Beginning with the isolated mandibular arch was a reasonable first step. To clarify this, we made an additional entry under our new heading in Results and Discussion: Study Limitations and Future Directions:

"● The present model represents an isolated mandible in space. Intuition suggests that occlusal loading from the maxillary arch probably plays some role in mandibular anterior arch changes, and a complete model redesign will be needed to study occlusal loading effects. By adding six maxillary anterior teeth to the present model (and including complex 3D surface-to-surface contact formulations) we have initiated a limited thesis study of the effects of anterior overbite increase on the mandibular anterior arch.

Dr. William Proffit stated that the major primary factors affecting dental equilibrium (or position imbalance and relapse) appears to be resting pressures of tongue and lips plus forces created within the periodontal tissues.6 The present, preliminary study is only one small, first step which corroborates Dr. Proffit’s thoughts and encourages future FEA relapse research. Beginning with the isolated mandible was a reasonable first step."

Thank you Dr. Esam Abdullah Alhendi for your comments and suggestions. They have strengthened our manuscript.

Attachment Submitted filename: Response to Reviewers_final.docx

# Peer review: Decision Letter 1

**DOI:** 10.1371/journal.pone.0353500.r003

**Authors:** Tanay Chaubal

13 Sep 2026

The effect of resting tongue pressure, lip-cheek pressure, and interdental force on mandibular arch collapse and mesial molar drift: A preliminary 3D finite element study

PONE-D-26-31696R1

Dear Dr. Steven Marshall,

We’re pleased to inform you that your manuscript has been judged scientifically suitable for publication and will be formally accepted for publication once it meets all outstanding technical requirements.

Within one week, you’ll receive an e-mail detailing the required amendments. When these have been addressed, you’ll receive a formal acceptance letter and your manuscript will be scheduled for publication.

An invoice will be generated when your article is formally accepted. Please note, if your institution has a publishing partnership with PLOS and your article meets the relevant criteria, all or part of your publication costs will be covered. Please make sure your user information is up-to-date by logging into Editorial Manager at Editorial Manager® and clicking the ‘Update My Information' link at the top of the page. For questions related to billing, please contact billing support.

If your institution or institutions have a press office, please notify them about your upcoming paper to help maximize its impact. If they’ll be preparing press materials, please inform our press team as soon as possible -- no later than 48 hours after receiving the formal acceptance. Your manuscript will remain under strict press embargo until 2 pm Eastern Time on the date of publication. For more information, please contact onepress@plos.org.

Kind regards,

Tanay Chaubal

Academic Editor

PLOS One

Additional Editor Comments (optional):

Reviewers' comments:

Reviewer's Responses to Questions

**Comments to the Author**

Reviewer #1: All comments have been addressed

Reviewer #2: All comments have been addressed

**********

2. Is the manuscript technically sound, and do the data support the conclusions?

Reviewer #1: Yes

Reviewer #2: Yes

**********

3. Has the statistical analysis been performed appropriately and rigorously?

Reviewer #1: Yes

Reviewer #2: I Don't Know

**********

4. Have the authors made all data underlying the findings in their manuscript fully available?

The PLOS Data policy

Reviewer #1: Yes

Reviewer #2: Yes

**********

5. Is the manuscript presented in an intelligible fashion and written in standard English?

Reviewer #1: Yes

Reviewer #2: Yes

**********

Reviewer #1: Wonderfull job, analysis is well done, explaining the effect of soft tissue on the arch is addressed clearly

Reviewer #2: Dear Editor,

I have reviewed the revised manuscript and the authors' detailed responses. Their scientific justifications regarding the model's limitations (such as PDL omission and material properties) are sound for a preliminary FEA study, and I appreciate their full compliance in modifying the title as requested. The revisions have addressed all key concerns effectively, and I fully recommend accepting the manuscript for publication in its current form

**********

what does this mean?). If published, this will include your full peer review and any attached files.

If you choose “no”, your identity will remain anonymous but your review may still be made public.

**Do you want your identity to be public for this peer review?**  For information about this choice, including consent withdrawal, please see our Privacy Policy

Reviewer #1: **Yes:**  Ahmed Abouelnour

Reviewer #2: **Yes:**  Esam Alhendi

**********

# Editor report: Acceptance letter

**DOI:** 10.1371/journal.pone.0353500.r004

**Authors:** Tanay Chaubal

PONE-D-26-31696R1

PLOS One

Dear Dr. Marshall,

I'm pleased to inform you that your manuscript has been deemed suitable for publication in PLOS One. Congratulations! Your manuscript is now being handed over to our production team.

At this stage, our production department will prepare your paper for publication. This includes ensuring the following:

* All references, tables, and figures are properly cited

* All relevant supporting information is included in the manuscript submission,

* There are no issues that prevent the paper from being properly typeset

You will receive further instructions from the production team, including instructions on how to review your proof when it is ready. Please keep in mind that we are working through a large volume of accepted articles, so please give us a few days to review your paper and let you know the next and final steps.

Lastly, if your institution or institutions have a press office, please let them know about your upcoming paper now to help maximize its impact. If they'll be preparing press materials, please inform our press team within the next 48 hours. Your manuscript will remain under strict press embargo until 2 pm Eastern Time on the date of publication. For more information, please contact onepress@plos.org.

You will receive an invoice from PLOS for your publication fee after your manuscript has reached the completed accept phase. If you receive an email requesting payment before acceptance or for any other service, this may be a phishing scheme. Learn how to identify phishing emails and protect your accounts at https://explore.plos.org/phishing.

If we can help with anything else, please email us at customercare@plos.org.

Thank you for submitting your work to PLOS One and supporting open access.

Kind regards,

PLOS One Editorial Office Staff

on behalf of

Dr. Tanay Chaubal

Academic Editor

PLOS One
